# Wildfire spreading prediction using multimodal data and deep neural network approach

**DOI:** 10.1038/s41598-024-52821-x

**Published:** 2024-01-31

**Authors:** Dmitrii Shadrin, Svetlana Illarionova, Fedor Gubanov, Ksenia Evteeva, Maksim Mironenko, Ivan Levchunets, Roman Belousov, Evgeny Burnaev

**Affiliations:** 1 Skolkovo Institute of Science and Technology, Moscow, Russia 121205; 2 Faculty of Computational Mathematics and Cybernetics, Moscow State University, Moscow, Russia 119899; 3 The National Crisis Management Center, EMERCOM of Russia, Moscow, Russia 109012

**Keywords:** Environmental sciences, Natural hazards

## Abstract

Predicting wildfire spread behavior is an extremely important task for many countries. On a small scale, it is possible to ensure constant monitoring of the natural landscape through ground means. However, on the scale of large countries, this becomes practically impossible due to remote and vast forest territories. The most promising source of data in this case that can provide global monitoring is remote sensing data. Currently, the main challenge is the development of an effective pipeline that combines geospatial data collection and the application of advanced machine learning algorithms. Most approaches focus on short-term fire spreading prediction and utilize data from unmanned aerial vehicles (UAVs) for this purpose. In this study, we address the challenge of predicting fire spread on a large scale and consider a forecasting horizon ranging from 1 to 5 days. We train a neural network model based on the MA-Net architecture to predict wildfire spread based on environmental and climate data, taking into account spatial distribution features. Estimating the importance of features is another critical issue in fire behavior prediction, so we analyze their contribution to the model’s results. According to the experimental results, the most significant features are wind direction and land cover parameters. The F1-score for the predicted burned area varies from 0.64 to 0.68 depending on the day of prediction (from 1 to 5 days). The study was conducted in northern Russian regions and shows promise for further transfer and adaptation to other regions. This geospatial data-based artificial intelligence (AI) approach can be beneficial for supporting emergency systems and facilitating rapid decision-making.

## Introduction

Global climate change has led to an increase in temperature, which in turn has raised the frequency and overall risk of wildfires worldwide^1^. Smoke exposure is among consequences of wildfires that have a significant impact on human health^2^. Soil erosion and slow vegetation recovery have been thoroughly studied to evaluate the total damage to the environment caused by wildfires^3^. Forest losses affect carbon balance on a global scale and these changes are often caused by human activities^4^. Due to all these reasons, significant efforts are being made to predict wildfire spreading, prevent it and mitigate the damage.

Contemporary scholarly research emphasizes four main components of wildfire risk management: fire prevention and mitigation, preparedness, response, and recovery phases^5^. In this study, our focus is on predicting wildfire spread using multimodal data to assist in mitigating fire events after they have occurred. This prediction can be based on geo-spatial information, land cover characteristics, and weather conditions. The main differences in current studies lie in the choice of algorithms and data sources. Data sources can vary in spatial resolution (ranging from meters to kilometers) and temporal resolution (ranging from hours to days), addressing different practical requirements. By combining satellite imagery with weather measurements, multimodal data can provide a more comprehensive understanding of fire behavior and aid in identifying high-risk areas prone to ignition and fire spread. While unmanned aerial vehicles are currently used for short-term and small-scale fire danger monitoring and assessment^6^, satellite data is more preferable for long-term predictions spanning several days and covering large-scale areas.

The earliest algorithms for wildfire spreading prediction such as the Canadian forest fire behavior prediction system (CFFBPS) are based on empirical and theoretical techniques and rules to model the spreading process^7^. However, in recent years, machine learning (ML) and deep learning (DL) approaches have been increasingly used to solve hazard forecasting problems based on remote sensing data^8^. These algorithms can be used both for wildfire ignition probability^9^ and fire spreading prediction. For instance, FireCast^10^ presented convolutional neural network (CNN) model trained on geospatial data, such as satellite imagery, elevation data, weather data, and historical fire perimeters to identify patterns associated with fire spread in certain environments to produce predictions of wildfire spread.

The quality of ML-based methods depends directly on the quality of the dataset. It is a challenging task to assemble a high-quality dataset to train a model for predicting the spread of fires. A number of studies are devoted to fuel characteristics estimation based on satellite observations for forest areas^11,12^. Produced maps can be further used as input features for predictive models development. Hout et al.^13^ presented an open data resource for further research in the field of forest fire forecasting. This dataset combines nearly a decade of remote sensing data, utilizing characteristics such as topography, weather, drought index, vegetation, and population density with historical fire records. Using this data set, the potential of deep learning approaches for predicting forest fires based on remote sensing data is shown.

Another approach for predicting fire spread is to train a model on simulated data, the trained model can further predict the spread of a forest fire. This approach allows the generation of arbitrarily large datasets with diverse initial data and features. Alliare et al.^14^ proposed approach that consists of a trained DNN used for regression. The network has a hybrid architecture to deal with 2D fields of environmental parameters and with scalar inputs. Training was carried out with a large dataset of size $5 \times 10^6$ and a complementary test sample of size $10^4$, the target model of fire propagation is obtained using the *ForeFire* numerical solver. The model showed satisfactory performance, explaining 94% of the variance of the output.

Bolt et al.^15^ presented an emulator based on deep neural network (DNN). It approximates the fire front and can be used in the future to more effectively characterize a wide range of fire scenarios simulated in various environmental conditions. Burge et al.^16^ demonstrated the efficacy of the ConvLSTM model on several corpuses of simulated data generated by an analogue model. This research shows that ConvLSTMs can capture local fire transmission events, as well as the overall fire dynamics, such as the rate at which the fire spreads.

The objective of this study is to propose the effective ML-based methodology for predicting fire spread that is able to cover vast territories taking into account the most important environmental parameters, that influence the direction and speed of fire spread. The forecasting horizon of 1 to 5 days in our study is purposefully selected to align with the availability of high-resolution meteorological data, which is crucial for accurate wildfire prediction. Longer-term forecasts are constrained by the current resolution of meteorological inputs. Furthermore, the primary aim of this work is to support immediate wildfire response efforts, necessitating highly accurate short-term predictions. Accuracy tends to diminish over longer periods due to the cumulative nature of prediction errors and the inherent unpredictability of environmental factors. One of the key questions addressed in the study is how to design an effective pipeline that combines proper data collection and neural network model training. The proposed study is mainly focused on large fires that spread on huge territories and may lead to serious ecological and economical consequences. We integrate and automatically process static features that describe the properties of the surface. In real-life monitoring systems, some data can be unavailable due to disruptions or noise in operational weather forecasts for some parameters. Therefore, it is crucial to estimate the importance of each input feature and the implications of its absence. Feature importance analysis is implemented in the study to better understand the process and further improve the model. The main advantage of the proposed ML-based approach in comparison with empirical approaches is its ability to be automatically scaled to vast territories by extended training dataset with new environmental examples. We validated the developed approach in three vast Russian regions to prove its effectiveness. The main contribution of the study is as follows:

- We designed a DL-based approach for fire spreading prediction.
- We analyzed geo-spatial features and their importance for model development.
- We shared the methodology of data collection and processing.

The paper is organized as follows: the section titled as “Methodology and data” section presents our proposed methodology and provides a description of the data utilized. The “Results” section showcases the outcomes obtained from our study, while the “Discussion” section focuses on analyzing and interpreting these findings. In the “Conclusion” section, we present our final remarks.

## Methodology and data

### Study area

**Figure 1:**
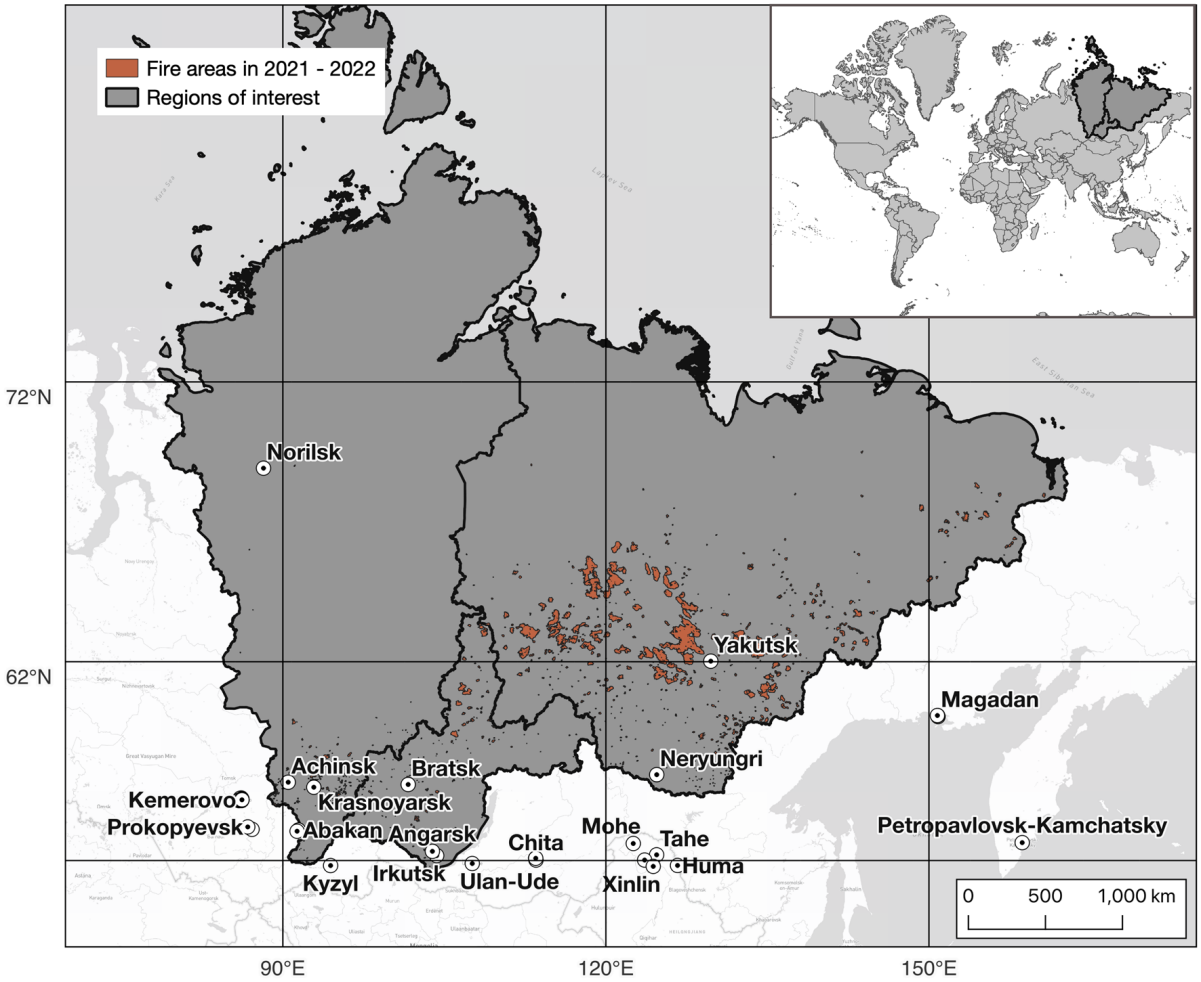
Study regions: Krasnoyarsk Territory, Irkutsk Region, Republic of Sakha. The map was generated with the QGIS v.3.14 software (https://qgis.org/en/site/).

The dataset for this study includes data for 2021–2022 years for the following regions of the Russian Federation: Krasnoyarsk Territory, Republic of Sakha (Yakutia), and Irkutsk Region (Fig. 1). In 2019, carbon emissions resulting from forest fires within these regions attained 82 million tons, and in the entire expanse of the Russian Federation—284 million tons^17^. Furthermore, as demonstrated in the research conducted by Romanov et al.^18^, the Republic of Sakha emerged as the primary contributor to PM_2.5_ emissions in Russia during the year 2021, accounting for a substantial 6.1 Mt out of the total 8 Mt of PM_2.5_ emissions within the country. This data underscores the significant contribution of these regions to the overall emissions from wildfires in the Russian Federation.

Krasnoyarsk Territory, Irkutsk Region, and the Republic of Sakha constitute significant portion of the Eastern Siberian geographic expanse spanning an area of 6,225,166 km^2^. The prevailing vegetative landscape is emblematic of the taiga biome, characterized by the conspicuous prevalence of coniferous woodlands, primarily composed of boreal species such as spruce, fir, larch, and pine. Eastern Siberia’s geographic domain encompasses a range of distinct climatic zones: to the south, there is an extracontinental climate; towards the central areas, a moderately continental climate prevails; and on the north the climate becomes strikingly continental, transitioning into subarctic and arctic climate regimes. Precipitation levels in Eastern Siberia are generally lower when compared to the western regions of Russia. The topographical attributes of the region of interest encompass vast plains, high plateaus, mountain ranges, and large river systems.

### Reference data

A total of 947 fires representing the subsequent classifications were selected as reference data: uncontrolled fire, forest fire, natural fire, and peat fire. Specifically, within this dataset, there were 183 fires recorded in the Irkutsk Region, 205 fires in the Krasnoyarsk Territory, and 559 fires in the Republic of Sakha.

**Figure 2:**
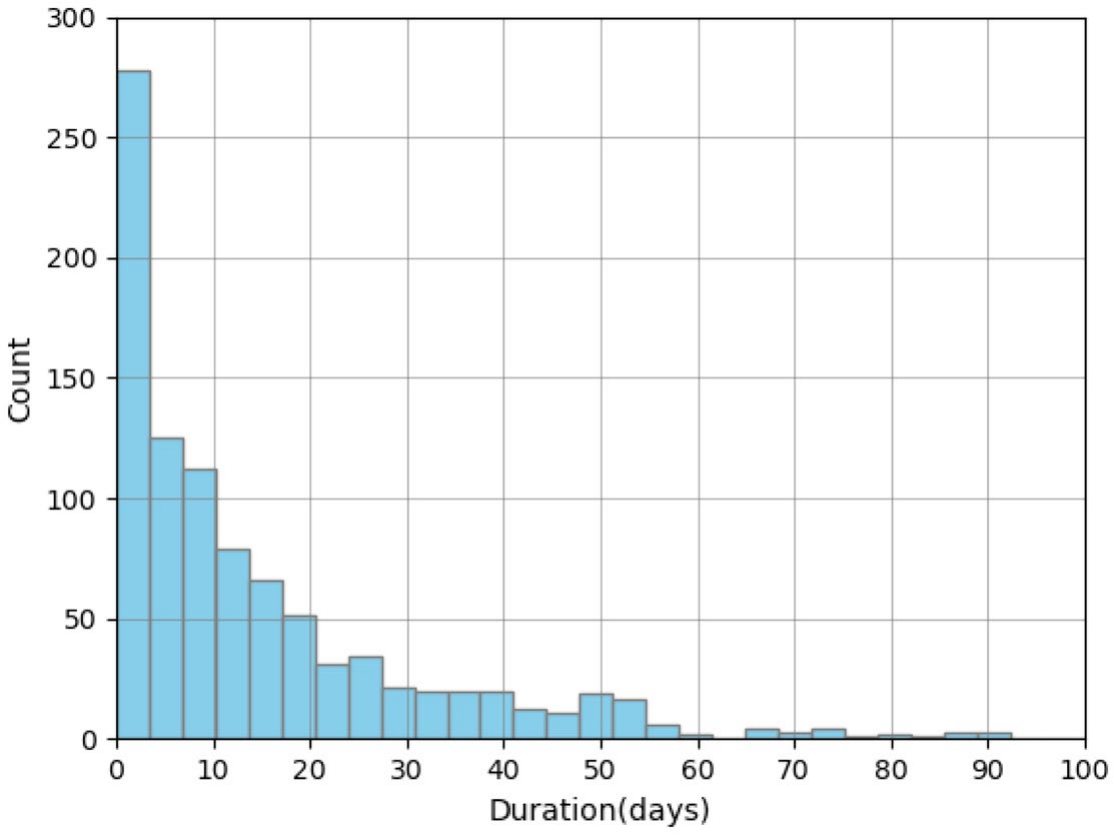
Distribution of fires duration.

The duration of fires varies from one day to three months, with 50% burning for less than ten days and 75% burning for less than twenty days (Fig. 2). The majority of fires last 2 days.

**Figure 3:**
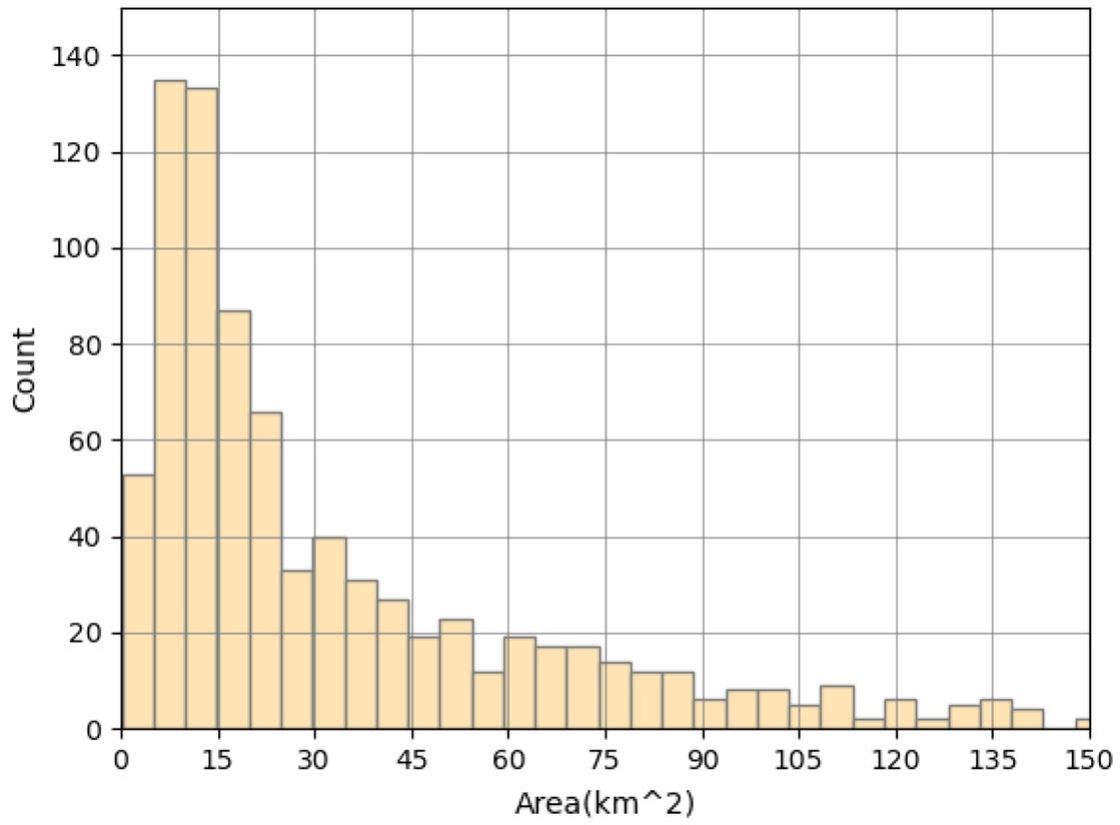
Distribution of burned areas.

Burning areas vary from 0.5 to 150 km^2^. About 50% of the fires burn in areas less than 30 km^2^ and more than 75% do not exceed 50 km^2^ (Fig. 3). Especially large areas burn in the Republic of Sakha.

**Figure 4:**
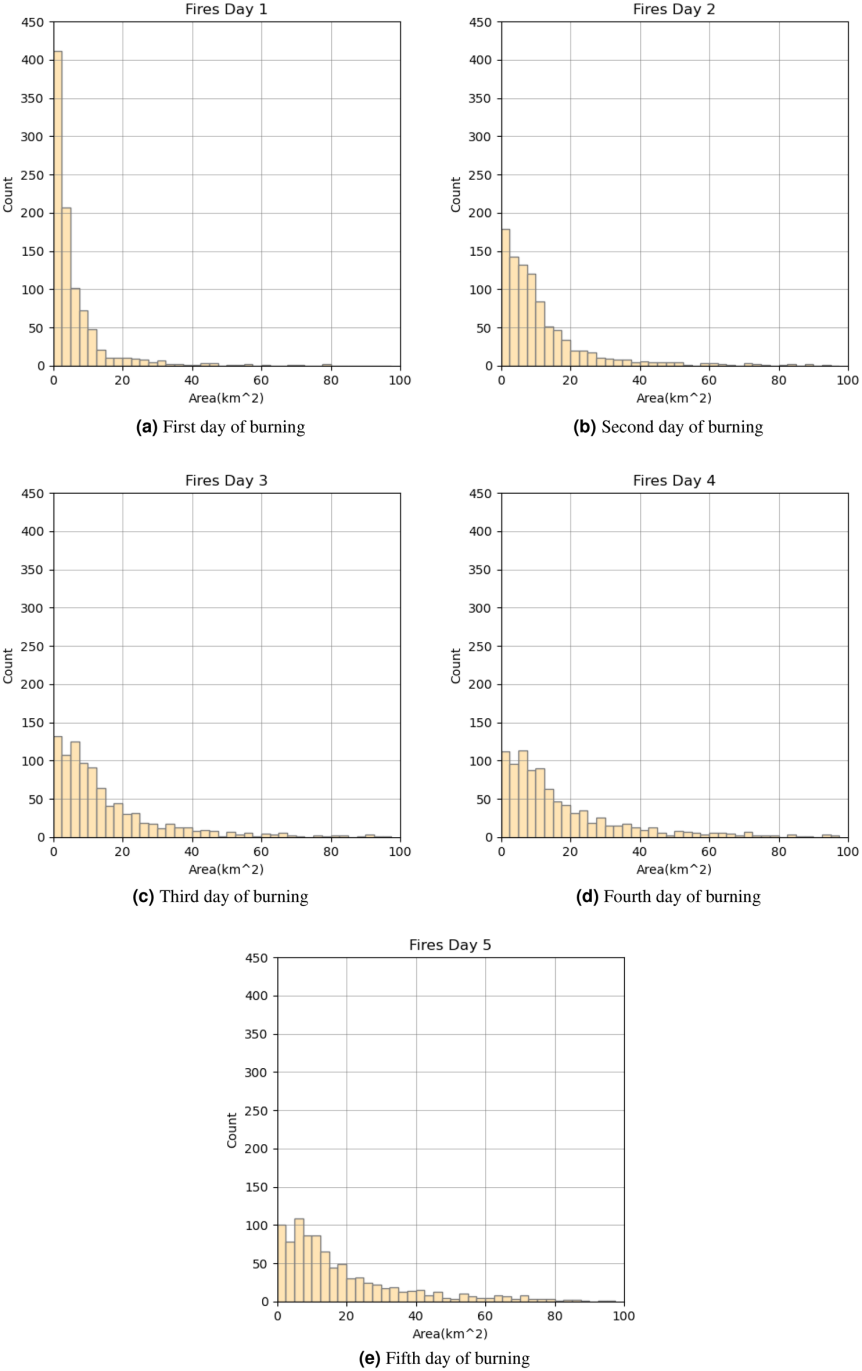
Distribution of burned areas for the first 5 days of burning.

Figure 4 shows the distribution of fire areas for the first 5 days of burning. There is an obvious pattern in that most fires did not have areas more than 20 km^2^. Due to this empirical observation, the determination was made to assess predictive domains of approximately 20 $\times$ 20 km around ignition center.

### Remote sensing and geospatial data

Each fire instance in the dataset was accompanied by precise ignition point coordinates and the corresponding fire initiation date. Utilizing these ignition point coordinates, the centroid of each fire was computed, and a bounding box was generated employing the geopandas.GeoDataFrame.buffer function^19^. The buffer diameter was determined to be approximately 21 × 21 km.

For each acquired region of interest, data retrieval was performed from open data sources. Table 1 presents comprehensive details pertaining to the employed datasets, including their spatial resolution, the period of data acquisition, the format and units in which the data was obtained.

**Table 1:** Open data sources used for dataset preparation.

Data source	Acquisition period	Spatial resolution	Units	Format	Description
MODIS MCD12Q1 v061^20^	2021 year	500 m	Classes	GeoTIFF	Land cover: annual International Geosphere–Biosphere Programme classification
Copernicus GLO-30 digital elevation model^21^	2011–2015 years	30 m	m	GeoTIFF	Elevation
WorldPop population density^22^	2020 year	30 arc-seconds	Number of people per km^2^	GeoTIFF	Population density
MODIS MOD15A2H v061^23^	8 days before fire initiation	500 m	%	GeoTIFF	Fraction of photosynthetically active radiation
			m^2^/m^2^		Leaf area index
MODIS MOD13A1 v061^24^	16 days before fire initiation	500 m	–	GeoTIFF	Normalized difference and enhanced vegetation index
MODIS MOD16A2 v006^25^	8 days before fire initiation	500 m	kg/m^2^	GeoTIFF	Evapotranspiration and potential evapotranspiration
MODIS MOD11A1 v061^26^	For every prediction day	1 km	K	GeoTIFF	Daytime and nighttime land surface temperature
ERA5-Land^27^	For every prediction day	9 km	m/s	NetCDF	Eastward and northward components of the 10 m wind
			K		Temperature in the atmosphere
			m		Accumulated liquid and frozen water, comprising rain and snow, that falls to the Earth’s surface

The acquired data underwent cropping based on the designated region of interest and was subsequently resized to dimensions of 128 $\times$ 128 pixels with a spatial resolution of 0.0059435° per pixel, employing bilinear interpolation. Furthermore, the weather data sourced from the ERA5-Land dataset underwent aggregation operations, encompassing computations for minimum and maximum values.

Subsequently, for each fire instance, the downloaded data was utilized to generate compressed archives in NPZ format, incorporating the following fields:

1. The “ignition” field was appended with an image with rasterized ignition points, denoting a value of “1” in pixels corresponding to ignition points and “0” in pixels representing the background.
2. Five images were integrated into the “static” field in the following sequence:
  - Land cover map for index “0”.
  - Elevation map for index “1”.
  - Aspect and slope maps, derived by applying the DEM (Digital Elevation Model) processing method from the GDAL library^28^ to the elevation map, for index “2” and “3” respectively.
  - Population density map for index “4”.
3. Furthermore, the “dynamic” field was enriched with 16 images for the fire initiation day, and 10 images were used for subsequent prediction days (refer to Table 2 for details).

**Table 2:** “Dynamic” field structure of an archive.

Image data	Field index in archives related to the fire initiation day	Field index in archives related to other prediction days
Fraction of photosynthetically active radiation	0	–
Leaf area index	1	–
Daytime land surface temperature	2	0
Nighttime land surface temperature	3	1
Normalized difference vegetation index	4	–
Enhanced vegetation index	5	–
Evapotranspiration	6	–
Potential evapotranspiration	7	–
Max value of eastward component of the 10 m wind	8	2
Max value of northward component of the 10 m wind	9	3
Max atmospheric temperature	10	4
Max precipitation	11	5
Min value of eastward component of the 10 m wind	12	6
Min value of northward component of the 10 m wind	13	7
Min atmospheric temperature	14	8
Min precipitation	15	9

**Figure 5:**
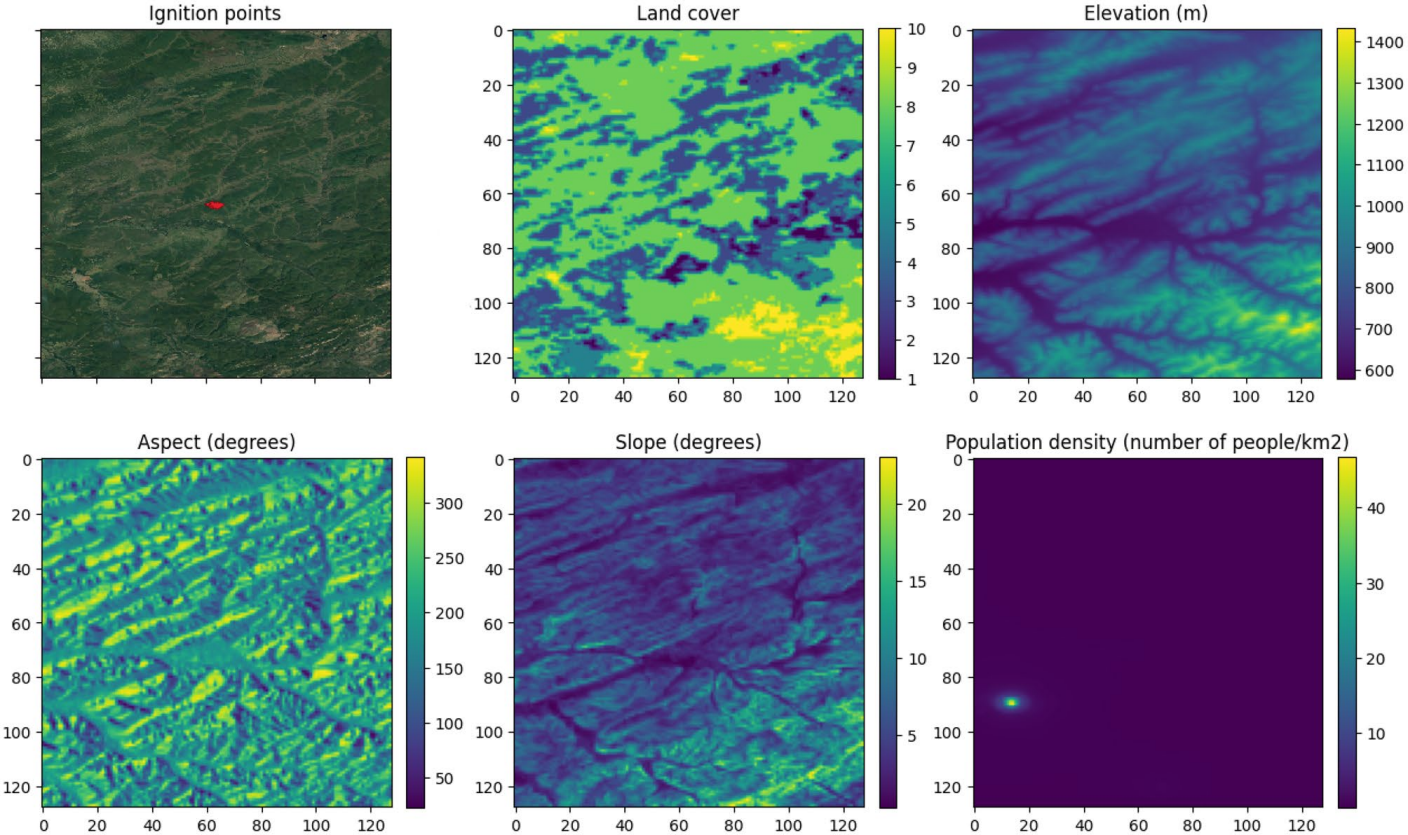
Example of ignition points and images in “static” field for them.

**Figure 6:**
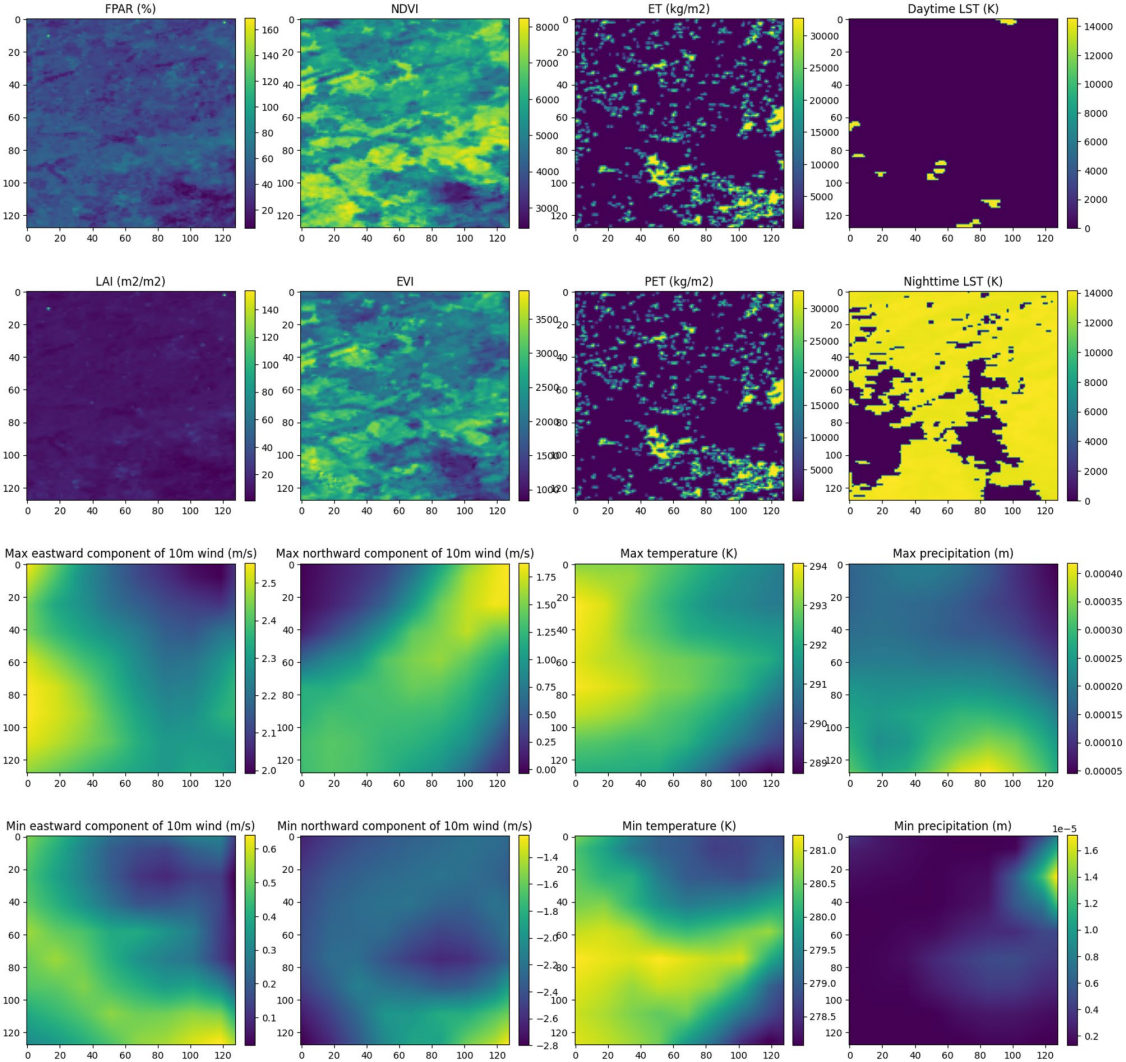
Example of images in “dynamic” field.

Figures 5 and 6 contain examples of images in “static” and “dynamic” fields of the compressed archives.

### Data preprocessing

The model receives as an input arrays data for each day. They contain static, dynamic features and ignition points. The arrays then go through the following processing steps:

- Feature values are set to [0, 1] by Min–Max normalization, and unknown values are set to − 1. Thus the input interval is given to [− 1, 1].
- Vegetation types are converted into binary masks for each class;
- Arrays with features have the size of 32 $\times$ 32 pixels (21 $\times$ 21 km).
- Arrays with features (layers) for all days are united in one multi-channel tensor.

As a result, we obtain a tensor with the following layers: 1 layer for ignition points on the first day, 21 static features, 6 vegetation features from MODIS, 10 dynamic weather features for each forecasting day. The total number of features for 5 days is 78. For a shorter forecasting period, we substitute 10 weather features for each day. Thereby, for 3 days, we have 58 features.

The dataset is divided into train and test sets in a ratio of 4:1. In the test set, fires for the Irkutsk region, the Krasnoyarsk region, and the Republic of Sakha were presented in the same ratio.

### Methodology

**Figure 7:**
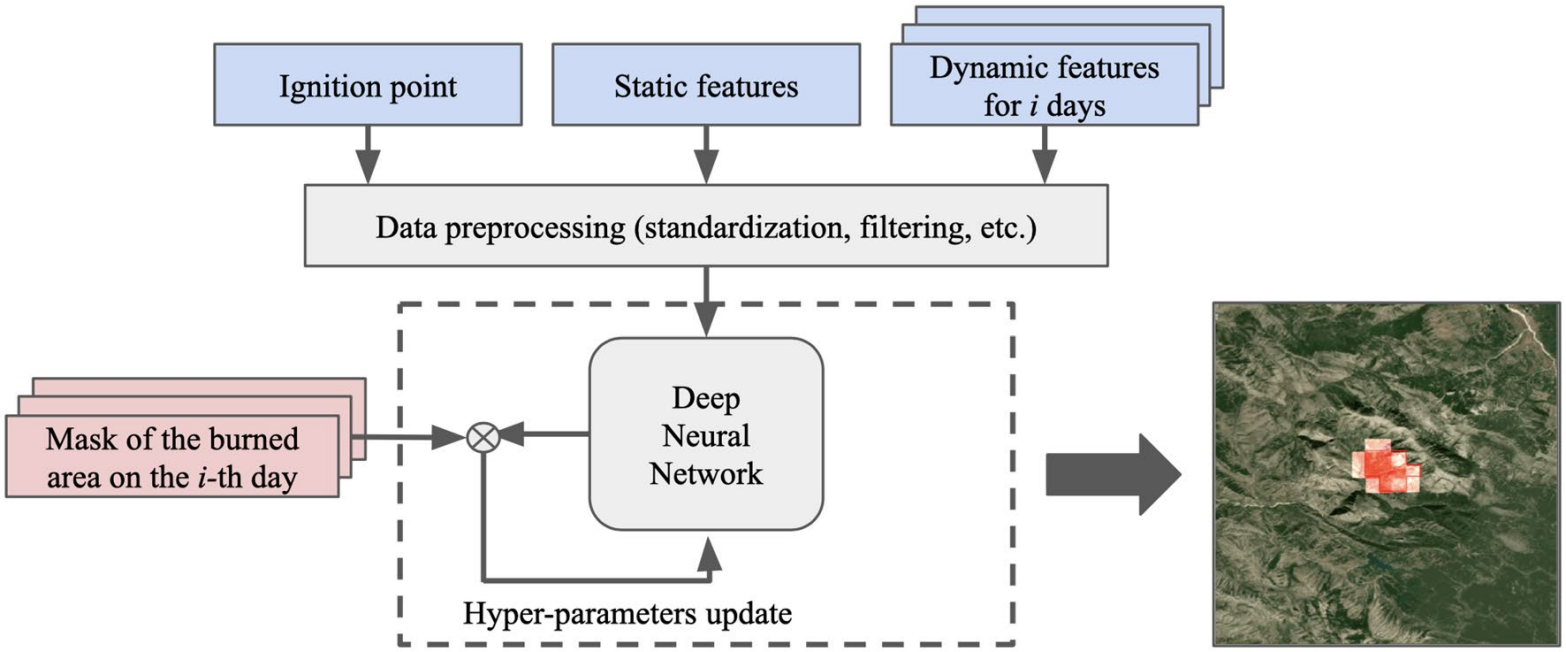
Study workflow. The map was generated with the QGIS v.3.14 software (https://qgis.org/en/site/), fire spreading example, and RGB satellite composite from Google Maps layers available in QGIS.

The proposed approach aims to estimate the direction and speed of the wildfire using spatial data, operational information about the fire ignition point, and forecast weather data. The scheme of the proposed approach is presented in Fig. 7. The features used, which are submitted to the model input, can be divided into three categories: fire points, static features, and dynamic features. Historical data were used to create a training dataset. We included a preprocessing step to filter the target fire events and bring all data within the required range of values for neural network training. The collected and preprocessed training examples were then used to tune the hyperparameters of the CNN. The model outputs a forecast of the burned area by fire on a given day (a mask of burned or burning area). Additionally, at the post-processing stage, the direction and speed of the wildfire spread were analyzed. The prediction horizon is 5 days, with a prediction step of 1 day. The forecasting horizon of 1 to 5 days in our study is purposefully selected to align with the availability of high-resolution meteorological data, which is crucial for accurate wildfire prediction. Longer-term forecasts are constrained by the current resolution of meteorological inputs. Furthermore, the primary aim of this work is to support immediate wildfire response efforts, necessitating highly accurate short-term predictions. Accuracy tends to diminish over longer periods due to the cumulative nature of prediction errors and the inherent unpredictability of environmental factors. The model estimates the spread of the fire on a grid with a spatial resolution of 650 m for each cell. The forecast area was set to 21 $\times$ 21 km, with the ignition point located in the center of this area. This modeling area size was chosen based on statistical analysis of the average burned area in 5 days. In general, wildfires do not spread more than 10 km in 5 days in the considered northern areas.

### Algorithms

In the experiment, popular architectures for segmentation task were utilized: U-Net^29^, U-Net++, MA-Net^30^, DeepLabV3^31^. The hyperparameters were uniformly defined for all models as follows: encoder backbone is ResNet18, number of stages used in encoder equals to 3, number of channels in decoder is (64, 32, 16) (except for DeepLab architectures), with default values for the other hyperparameters. The same dataset was used for training and validation across all experiments. Upon completion of training, metrics such as F1-score, Precision, Recall, IoU were compared, and a visual analysis of predictions was conducted. The conducted experiments included a comparative evaluation of mentioned architectures, along with an array of loss functions such as BCE loss, Dice loss, Focal loss, the combination of Dice and Focal loss, General Dice loss, and Tversky loss. The F1-score metric used to assess each model’s performance reveals variances in effectiveness with scores from 0.62 to 0.67, indicating no singularly superior loss function for all architectures.

In the subsequent stage, a list of loss functions was identified for comparison in various experiments with the MA-Net architecture. This list included primary loss functions used in segmentation tasks: BCEWithLogits Loss, Dice and Focal Loss, Focal Loss^32^, Generalized Dice Loss, and TverskyLoss^33^. We tested different hyperparameters of the MA-Net model, such as learning rate, batch size, special attention was given to compare loss functions: the speed of loss convergence, uniformity of training, final metrics on the validation set, and visual assessment of predictions.

MA-Net is a U-Net similar model to solve the image segmentation problem that includes two main components: positional attention block (PAB) and multiscale attention block (MFAB). Positional Attention Block (PAB) records spatial relationships between pixels in the global view, this block applies to the output of the Encoder model and allows the decoder to focus on specific areas of the feature map. The Multiscale Attention Block (MFAB) fixes channel dependencies between any object map by means of a multiscale semantic union of objects, this block is applied at each stage of decoder.

The MA-Net model has the depth of three (three blocks of Encoder–Decoder) pretrained on Imagenet dataset. The number of input channels of corresponding decoder layers are (64, 32, 16), number of channels for PAB unit in decoder equals to 64.

We used Adam optimizer with L2 = 1e−5 and learning rate equal to 3e−3. The learning rate was decreased during training according to the OneCycleLR^34^ rule. The model was trained for 35 epochs, with a batch size of 32. Early stopping was applied to prevent model overfitting.

We utilized a combination of Dice and Focal loss functions as our loss function. The weights assigned to the loss function were adjusted to penalize fire pixels that were further away from the points of ignition more heavily, while giving less penalty to fire pixels concentrated near the points of ignition. This approach promotes greater diversity in model predictions and enhances the model’s ability to accurately predict large fires.

### Evaluation metrics

To evaluate model performance, we use F1-score and Intersection Over Union (IoU) with micro-imagewise averaging. The metrics are computed for each image and then averaged for all samples in the test set.

Mean absolute error (MAE) and mean absolute percentage error (MAPE) metrics are considered both for area values and for propagation velocities in four directions (north, south, west, east). To calculate the propagation rate, the distance from the center of the ignition points to the edge of the projection mask in four directions is taken, multiplied by the pixel size and divided by the number of forecast days. To calculate the approximate area, the number of pixels of the projection mask is summed and multiplied by the area of one pixel.

For MAE and MAPE metrics, the number of pixels corresponding to the burnt area for the predicted and true masks, as well as their intersection, are calculated. Metrics are calculated using the following formulas:

$$\begin{aligned} MAE(N_{\text {pred}}, N_{\text {gt}})= & {} \left| N_{\text {pred}}-N_{\text {gt}}\right| *{\text {pixel} \_ \text {size}}^2,\\ MAPE(N_{\text {pred}}, N_{\text {gt}})= & {} \frac{\left| N_{\text {pred}}-N_{\text {gt}}\right| }{N_{\text {gt}}} \times 100\%. \end{aligned}$$

For MAE and MAPE metrics, let’s define $D_{\text {gt}}$ and $D_{\text {pred}}$ as ground truth and predicted velocity of propagation in each direction, respectively:

$$\begin{aligned} MAE(D_{\text {pred}}, D_{\text {gt}})= & {} \left| D_{\text {pred}}-D_{\text {gt}}\right| \\ MAPE(D_{\text {pred}}, D_{\text {gt}})= & {} \frac{\left| D_{\text {pred}}-D_{\text {gt}}\right| }{ (D_{\text {gt}} + \{0, \text { if } D_{\text {gt}} > 0, \text { else } D_{\text {pred}} \}) } \times 100\%. \end{aligned}$$

## Results

We compared different neural network architectures in the task of fire spreading forecasting for 3 days. The obtained results for six various loss functions are shown in Table 3. The highest F1-score is achieved for the MA-Net architecture with Dice + Focal loss. The next experiments are conducted with the MA-Net architecture.

The achieved results for the MA-Net model with the best configuration is presented in Table 4. For each forecasting horizon, we trained a separate model and calculated a number of metrics to analyze the quality of fire spreading prediction. For each forecasting horizon, we defined the best loss function based on F1-score on validation subset. Achieved results are presented in Table 5. The best F1-score (0.68) has been achieved for the first day of the prediction using the Tversky loss function. For the third day, it slightly decreases and equals to 0.67. For the fifth day, the quality equals to 0.65. While F1-score and IoU are one of the most commonly used metrics for remote sensing semantic segmentation tasks, we also considered MAE and MAPE metrics. They provide us better understanding and interpretable values for burned area. The absolute error of the predicted burned area is increased from 9.54 to 28.2 sq km for the first and the fifth day, respectively. Such an increase in MAE is connected with wider burned area for later days. Therefore, we should notice that the MAPE metric provides more intuitive measurements of model’s performance. MAPE for the first day equals to 22.8, while the metric for the fifth day is 24.1. Therefore, the achieved metrics show perspective for future integration of the model in a natural disasters monitoring system.

**Figure 8:**
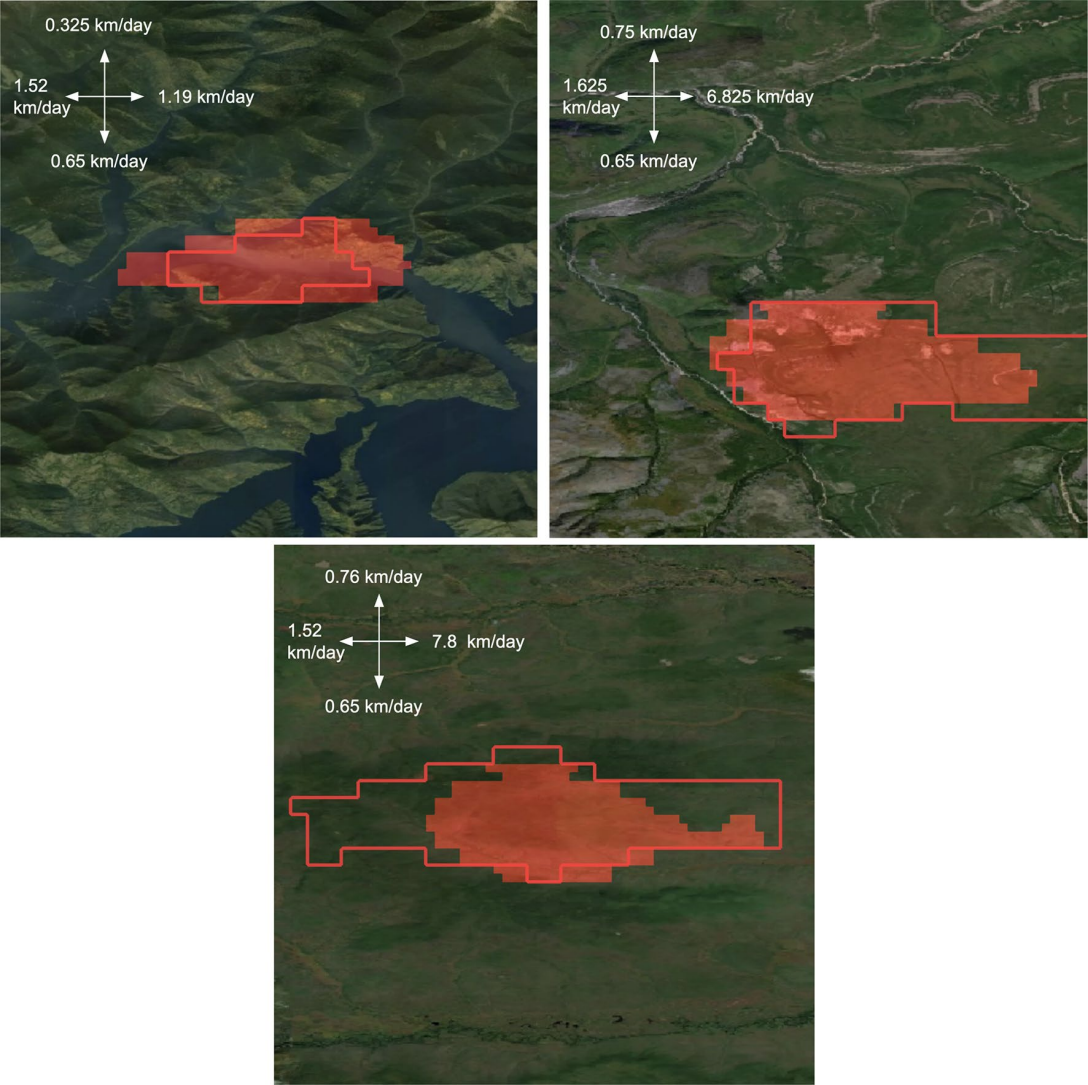
Example of model predictions for three days on the test set (MA-Net model). The red boundary is for ground truth fire perimeter, the red area is for model’s prediction. The map was generated with the QGIS v.3.14 software (https://qgis.org/en/site/) and RGB satellite composite from Google Maps layers available in QGIS.

**Figure 9:**
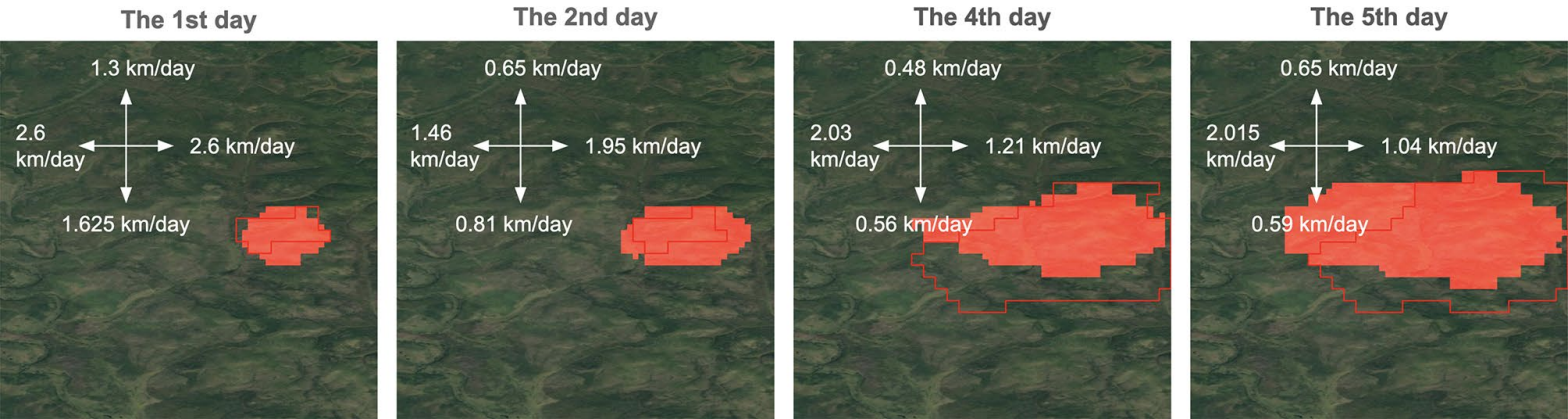
Example of model predictions for different days on the test set (MA-Net model). The red boundary is for ground truth fire perimeter, the red area is for model’s prediction. The map was generated with the QGIS v.3.14 software (https://qgis.org/en/site/) and RGB satellite composite from Google Maps layers available in QGIS.

For visual assessment, Fig. 8 shows model’s prediction for the third day for some test wildfire events. The model tries to fill the burned area. Predictions for other days are presented in Fig. 9. Although we did not set a goal to train model to predict the velocity of wild fire spreading in each direction, we computed it as a part of the post-processing. The achieved results are presented in Table 6. For the first day the average value for the four directions (north, south, west, east) equals to 0.74 km/day. It is suggested to add wild fire spreading direction as an additional component in loss function to adjust both the direction and area quality of the developed CNN model.

**Table 3:** The F1-score for different architectures depending on loss functions (Day 3).

	U-Net	U-Net++	MA-Net	DeepLabV3
BCE loss	$0.63 \pm 0.007$	$0.63 \pm 0.004$	$0.63 \pm 0.006$	$0.64 \pm 0.005$
Dice loss	$0.65 \pm 0.005$	$0.66 \pm 0.007$	$0.65 \pm 0.007$	$0.66 \pm 0.006$
Focal loss	$0.62 \pm 0.006$	$0.64 \pm 0.004$	$0.63 \pm 0.004$	$0.63 \pm 0.006$
Dice + focal loss	$0.66 \pm 0.004$	$0.65 \pm 0.004$	$0.67 \pm 0.003$	$0.65 \pm 0.005$
General dice loss	$0.65 \pm 0.007$	$0.66 \pm 0.005$	$0.66 \pm 0.005$	$0.66 \pm 0.004$
Tversky	$0.65 \pm 0.005$	$0.66 \pm 0.006$	$0.66 \pm 0.006$	$0.66 \pm 0.005$

**Table 4:** Metrics for different days to evaluate area (MA-Net model).

	Day 1	Day 2	Day 3	Day 4	Day 5
F1-score	0.68	0.66	0.67	0.64	0.65
IoU	0.54	0.52	0.52	0.5	0.52
MAE	9.54	15.8	19.7	27.16	28.2
MAPE	22.8	25.3	25.9	24.6	24.1

The MAE metric is in sq km.

**Table 5:** The F1-score metrics for different forecasting horizon depending on loss function (MA-Net model).

	Day 1	Day 2	Day 3	Day 4	Day 5
BCE loss	0.64	0.58	0.63	0.65	0.57
Dice + focal loss	0.65	0.65	0.67	0.64	0.62
General dice loss	0.66	0.66	0.66	0.62	0.64
Tversky	0.68	0.64	0.66	0.65	0.65

**Table 6:** Metrics for different days to evaluate fire spreading direction.

	Day 1	Day 2	Day 3	Day 4	Day 5
MAE					
$\text {North}$	0.55	0.34	0.29	0.25	0.2
$\text {South}$	0.49	0.29	0.32	0.26	0.19
$\text {West}$	1.01	0.82	0.55	0.49	0.35
$\text {East}$	0.91	0.70	0.51	0.48	0.34
MAPE					
$\text {North}$	54.9	60.6	69.2	69.7	62.6
$\text {South}$	48.5	46.07	57.9	62.3	54.9
$\text {West}$	47.5	52.25	53.1	64.2	51.5
$\text {East}$	59.7	69.9	61.8	87.9	60.3

The MAE metric is in km/day (MA-Net model).

### Feature importance

The input of the model consists of a multichannel tensor with a spatial resolution of 21 $\times$ 21 km. It includes 11 static features, such as the vegetation map, represented by 17 binary arrays, as well as 10 dynamic features for each of the 5 days. While some attributes may have clear relevance, the significance of others may be uncertain. Therefore, evaluating the importance of features can aid in reducing the feature space and gaining a deeper understanding of the model’s functioning.

One possible approach to assessing significance is by learning a model on different sub-samples of traits and observing changes in basic metrics. Although this approach is meaningful, it can be computationally inefficient. Our approach to assessing significance involved inferring the model on different sub-samples of validation features. More precisely, we zeroed out the values of the selected feature in all validation sample examples. Then, we used the trained model to make predictions on the modified data and compared the resulting metrics with the reference. This approach enables us to estimate the model’s dependence on specific features without requiring retraining of the model.

**Table 7:** Feature importance estimation based on feature groups. Certain feature groups are exclude and model’s performance is estimated for the third fire spreading day.

	IoU	F1-score	MAE
All features are included (forecast for the third day)	0.529	0.664	19.7 sq km
Land cover, NDVI, EVI	− 10%	− 6.3%	+ 33.3%
Fraction of PA radiation absorbed by green vegetation, LAI	− 8.3%	− 5.3%	+ 16.9%
DEM, average aspect and slope	− 4.3%	− 1.8%	+ 4.1%
Daytime and nighttime land surface temperature	− 3%	− 1.1%	+ 8.2%
Daytime and nighttime land surface temperature, air temperature at a height of 2 meters (min, max)	− 5.1%	− 2.8%	+ 9.5%
Actual and potential evapotranspiration	− 3.8%	− 1.7%	+ 11.5%
The eastern component of the wind (min, max)	− 43.9%	− 36.6%	+ 401.2%
North wind component (min, max)	− 42.7%	− 35.7%	+ 73.7%
Total precipitation (min, max)	− 1.3%	+ 0.3%	− 0.6%
Actual and potential evapotranspiration, total precipitation (min, max)	− 3.6%	− 1.5%	+ 11%

**Figure 10:**
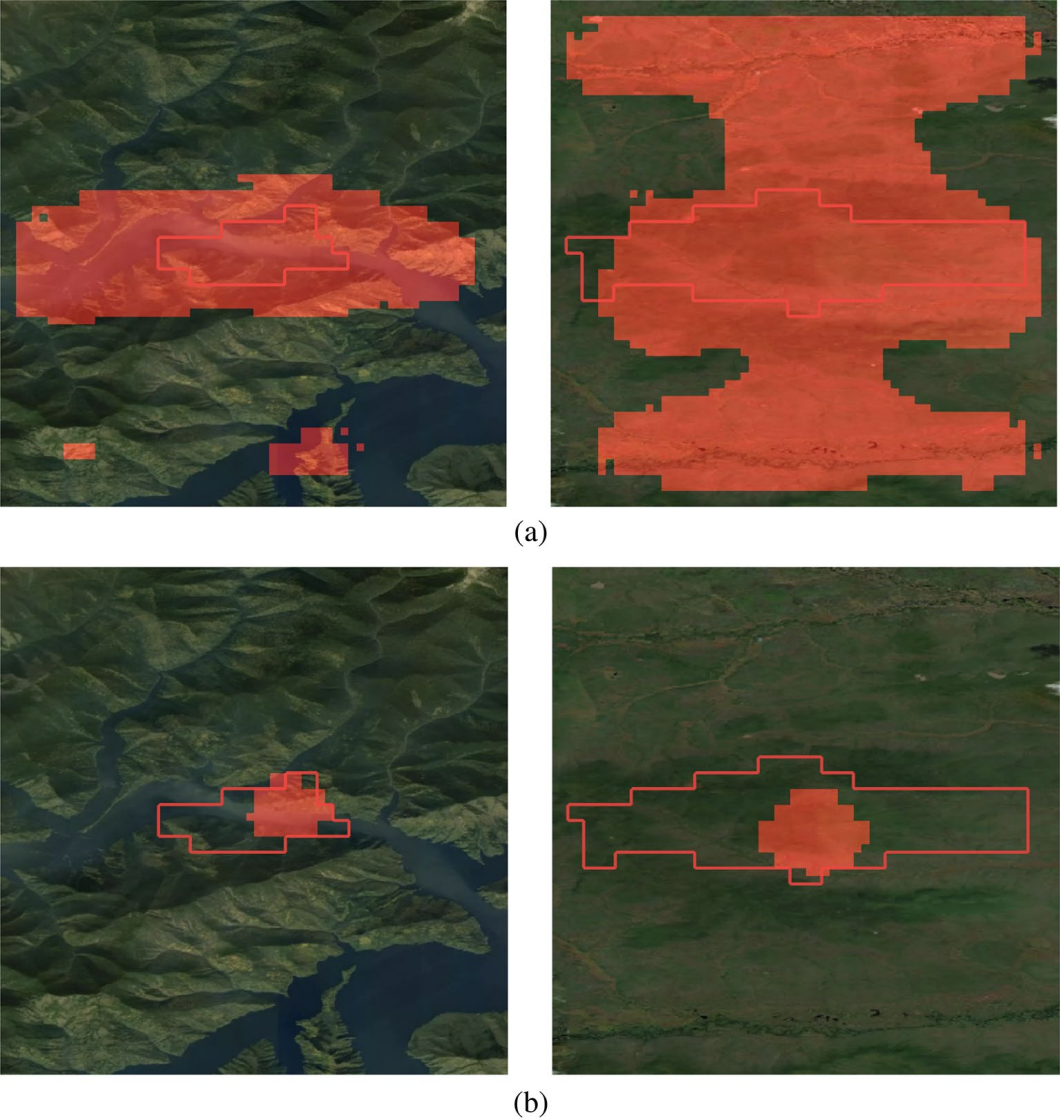
Example of feature importance: exclusion of the east wind component (**a**) and the north wind component (**b**). The red boundary is for ground truth fire perimeter, the red area is for model’s prediction. The map was generated with the QGIS v.3.14 software (https://qgis.org/en/site/) and RGB satellite composite from Google Maps layers available in QGIS.

Results of feature importance analysis is presented in Table 7. It is worth noting that there is a correlation between some features (NDVI and EVI, north- and east-components of wind, elevation, slope, aspect) so that groups of features were evaluated separately. When we exclude the group of features representing land cover properties, the F1-score decreases from 0.66 to 0.589. Exclusion of wind characteristics leads to the F1-score of 0.51. Figure 10 shows model’s prediction when particular features are excluded. We can see the importance of proper wind measurements for the trained model. When this information is absent, the model fails to provide an accurate prediction of fire spreading.

## Discussion

The task of forecasting fire spread has undergone numerous changes and improvements over the past decades. This evolution has been driven by the variety of approaches, methods, and technologies employed by researchers in the field. It is worth noting that different approaches can vary from the collection and processing of data to the final post-processing of predictions.

The primary focus in this section of the discussion is to compare our approach with other methods presented in academic literature. We will consider the specificity of data usage, architectural decisions, as well as the peculiarities of the training process to identify key differences and potential advantages of our methodology in comparison with others.

Radke et al.^10^ set the primary goal that was accurate prediction of the spreading of the fire perimeter in the near future, utilizing a high-resolution visual data (30 m resolution) and weather data (3 km resolution) for the next 24 h following a given burn perimeter. The model makes pixel-wise predictions based on data for a square area of 30 pixels around the concerned pixel. Our work aimed at forecasting the spread several days ahead using lower resolution data (650 m per pixel).

Hout et al.^13^ adjusted data to a 1 km resolution. Comparing features like topography, vegetation, and weather at 1 km and 650 m resolution, they show no significant difference. However, significant variation is seen when comparing fire masks at these resolutions, particularly for fires less than 5 km^2^. Given the prevalence of ’small’ fires ($< 5\,{\text {km}}^2$) in the dataset, training an accurate prediction model becomes more complex. The data preprocessing suggestion is to use the previous day’s fire mask as an input feature, which could lead the model to consistently predict a similar perimeter to the previous day’s, due to the often uneven spread of fires, when the fire perimeter remains unchanged for several days.

The main characteristic of these studies^14–16^ is the employment of fire simulations under defined conditions, making use of various mathematical analog models to generate fire scenarios. This approach has a twofold benefit. On the one hand, it facilitates the creation of a comprehensive dataset, encapsulating a wide range of feature combinations that could be instrumental in understanding and predicting fire behaviors under different circumstances. This enriched dataset can be a valuable asset for training and testing predictive models, offering a broad spectrum of scenarios for analysis. On the other hand, there is a significant drawback as this method tends to overlook the inherent noise and unpredictability present in real-world data. Real fires are influenced by a myriad of factors, some of which can be highly unpredictable or not well understood, thus they might not be well-represented in a simulated environment. The discrepancy between mathematically simulated fires and real fires could potentially lead to models that are theoretically sound but might perform poorly when confronted with real-world, noisy data. Therefore, real-world datasets with verified wildfire events are highly valuable and can be further combined with simulated data. Moreover, the focus in most studies is predominantly on short-term predictions, extending up to a few hours that is crucial for fire-risk management near human settlements^35^. While the objective of our work was to forecast fires spreading over several days for long-term planning on a large-scale. The primary aim of our study was to forecast fire spread over several days for long-term planning on a large scale in contrast to the common focus of most studies on short-term predictions of up to a few hours, which are crucial for immediate fire-risk management near human settlements^35^.

### Water objects

**Figure 11:**
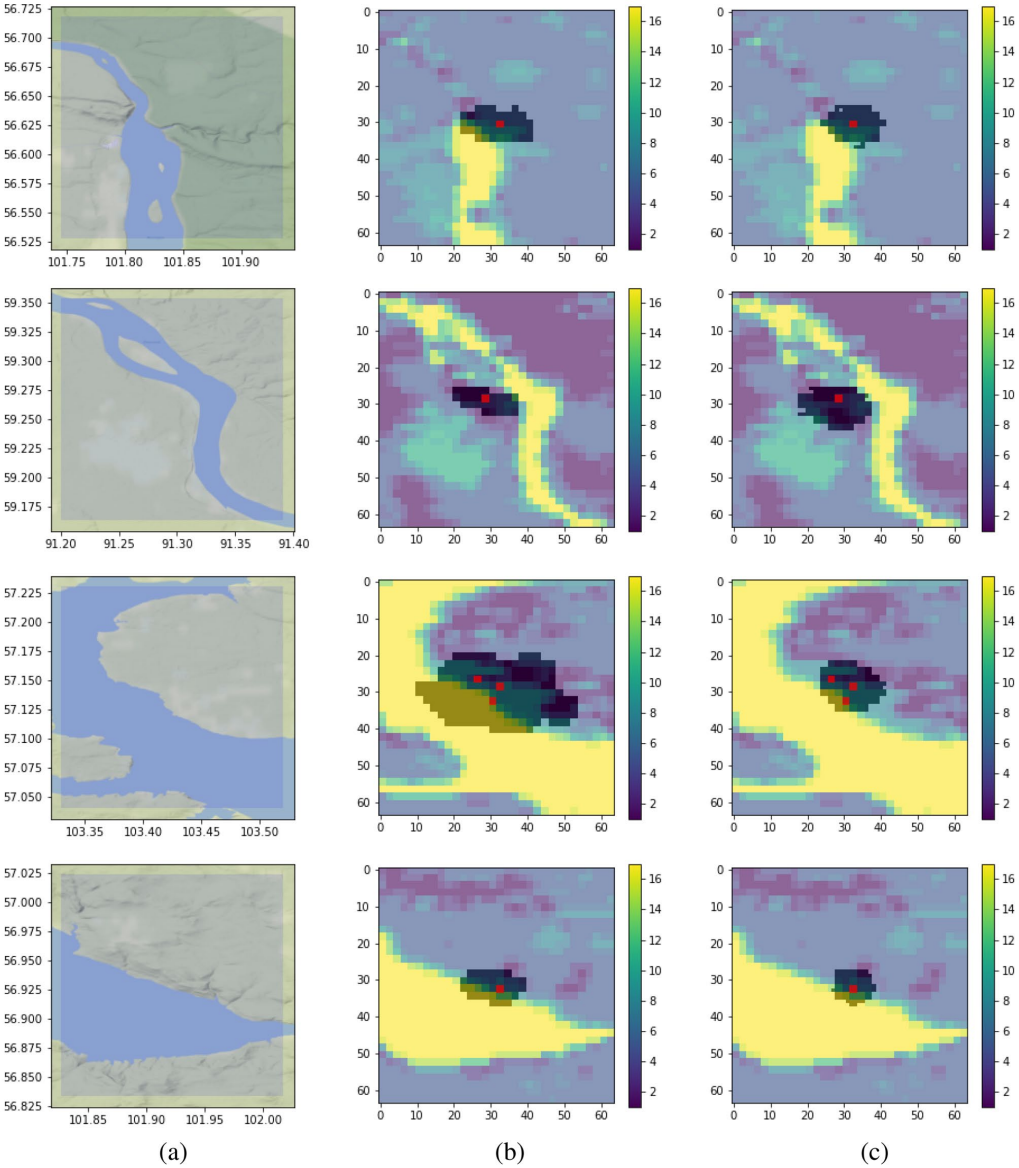
Fire spreading predictions near water bodies. Study area (**a**), reference masks with burned area (**b**), model predictions (**c**). Red points depict ignition points. Yellow areas in sub-figures (**b,c**) referee to water bodies class from the MODIS product.

Model’s ability to take into account water bodies is another important issue for discussion. We analyzed its behavior near large lakes and rivers, and the experimental results are presented in Fig. 11. In Fig. 11, the target fire masks are overlaid with the land cover map that contains the water mask. It is interesting to note that ground truth masks with fire spreading have a specific characteristic that can be explained by the nature of the process. The burned area is defined based on the temperature anomaly of the surface. At a short distance from the bank of a river or lake, the temperature might be significantly higher due to sparks and hot wind spread from the fire towards the water body. Thus, we occasionally observe rare ground truth masks that cover the water surface. However, due to a statistically sufficient amount of training examples of fire events, the model has learned the fire behavior near water bodies and produces predictions that do not cover the water surface. Currently, water body masks are derived from MODIS products. Such masks can be also produced with higher spatial resolution based on Sentinel-1 and Sentinel-2 images^36^. However, it assumes that other spatial features should be also brought to higher spatial resolution. The main limitation in this case is the weather forecast.

### Limitations and future work

**Figure 12:**
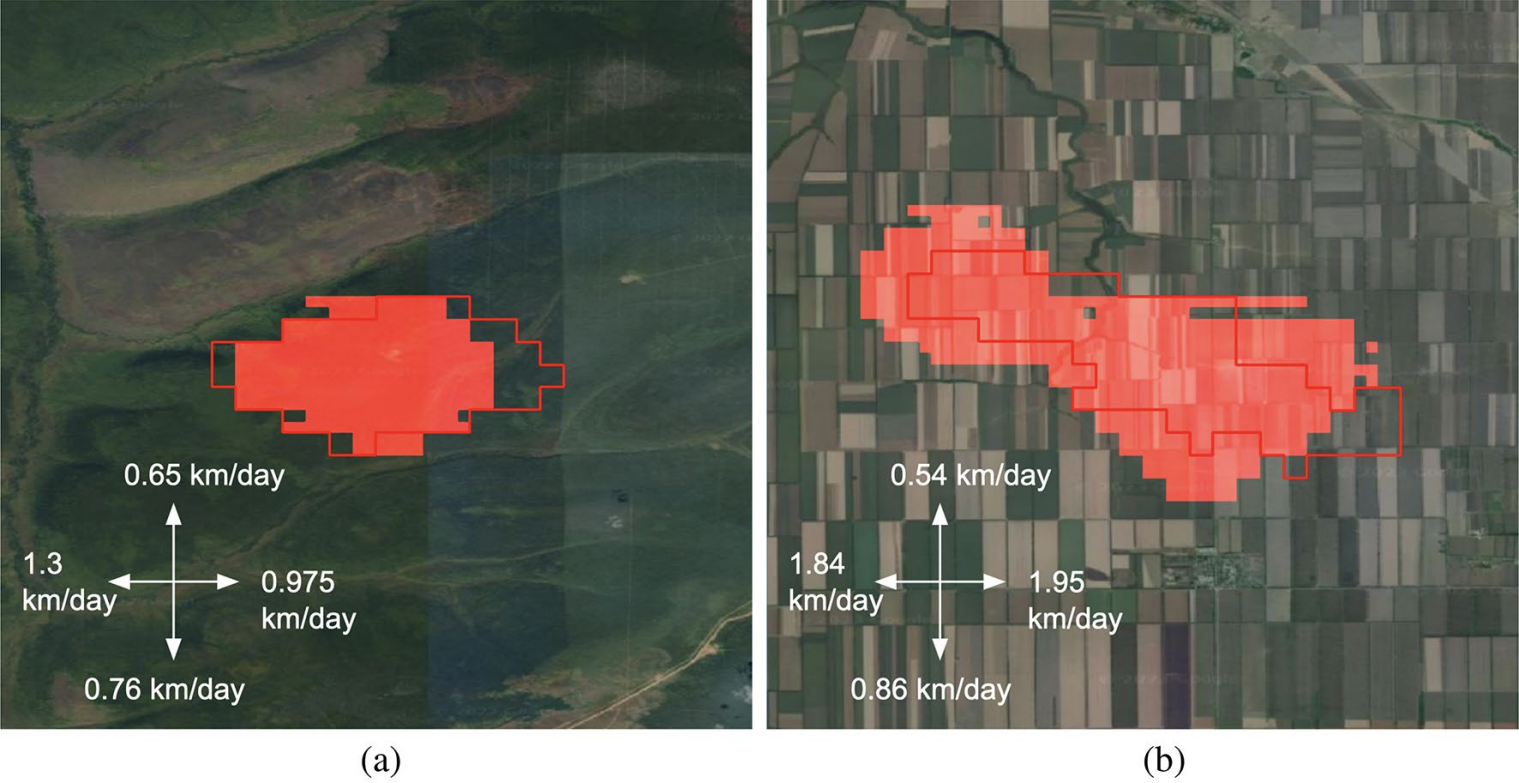
Example of model prediction for new regions: Irkutsk region (**a**), Rostov oblast (**b**). The red boundary is for ground truth fire perimeter, the red area is for model’s prediction. The map was generated with the QGIS v.3.14 software (https://qgis.org/en/site/) and RGB satellite composite from Google Maps layers available in QGIS.

We additionally checked the developed approach for other regions. Figure 12 depicts a compression between wildfire in Rostov oblast and Irkutsk region for the third day of fire events. Although this test sample for Irkutsk region was not in the training subset, the model’s prediction is rather accurate. The F1-score is 0.85, the IoU is 0.74. The area metrics such as MAE and MAPE are also high and equal to 3.9 sq km and 20%, respectively. However, for the fire event in Rostov region metrics are lower and equal to 0.65 and 0.48 for F1-score and IoU, respectively. MAE and MAPE are 19 sq km and 54%. One of the reasons in the prediction quality degradation is new environmental conditions for this south region. The model has been trained for north regions and learned patterns in landcover and weather properties. Therefore, we suggest additional model training for new regions with principle differences in environmental states.

It is widely recognized that detailed fuel maps play a significant role in predicting the occurrence and spread of wildfires^37^. Therefore, an encouraging direction for future research would be to integrate land cover and land use maps with higher spatial resolution^38^ into the fire spreading prediction pipeline. The spatial resolution of weather forecasting data is a limitation in the presented approach when more detailed fire spreading maps are required. However, there are studies that have proposed methods for weather forecast super-resolution^39^, which could be considered to achieve more precise results.

## Conclusion

Forecasting the spread of wildfires is an important task in fire management and mitigation. Satellite data has emerged as a promising source of information for this purpose. However, utilizing Earth remote sensing data effectively requires the development of new algorithms and machine learning approaches for spatial data processing and analysis. In this study, a unique dataset comprising Earth cover characteristics and meteorological measurements was collected and processed. The dataset encompasses several regions of the Russian Federation and includes verified fire data spanning multiple years. Additional variables were derived from satellite monitoring data, and a training dataset was created. A methodology was proposed to train a neural network algorithm based on the MA-Net architecture, enabling the prediction of fire spread up to 5 days in advance. Furthermore, additional characteristics such as the speed and direction of the fire front were determined. Analysis of the feature’s significance revealed that meteorological measurements are the most crucial factor in predicting fire spread. The model achieved an accuracy of F1-score 0.67 during testing for three days. The developed approach shows promise for further implementation in emergency monitoring systems, facilitating rapid analysis and faster decision-making to combat fires.

## Data Availability

The datasets used and analysed during the current study available from the corresponding author on reasonable request.
